# Identification of prognostic long non-coding RNA signature with potential drugs in hepatocellular carcinoma

**DOI:** 10.18632/aging.203322

**Published:** 2021-07-20

**Authors:** Fengjie Hao, Nan Wang, Xiang Wang, Yongjun Chen, Junqing Wang

**Affiliations:** 1 Department of General Surgery, Ruijin Hospital, Shanghai Jiao Tong University School of Medicine, Shanghai, P.R. China; 2 Department of Immunology, Ophthalmology and ORL, Complutense University School of Medicine, Madrid, Spain; 3 12 de Octubre Health Research Institute (imas12), Madrid, Spain; 4 Department of Quantitative and Computational Biology, Baylor College of Medicine, Houston, TX 77030, USA

**Keywords:** hepatocellular carcinoma, long non-coding RNA, prognosis, drug response

## Abstract

Hepatocellular carcinoma (HCC) is the primary malignancy in the liver with high rate of death and recurrence. Novel prognostic model would be crucial for early diagnosis and improved clinical decision. The study aims to provide an effective lncRNA-based signature to predict survival time and tumor recurrence for HCC. Based on public database, lncRNA-based classifiers for overall survival and tumor recurrence were built with regression analysis and cross validation strategy. According to the risk-score of the classifiers, the whole cohorts were divided into groups with high and low risk. Afterwards, the efficiency of the lncRNA-based classifiers was evaluated and compared with other clinical factors. Finally, candidate small molecules for high risk groups were further screened using drug response databases to explore potential drugs for HCC treatment.

## INTRODUCTION

Hepatocellular carcinoma (HCC) is the most common primary malignancy in the liver and the seventh most frequent neoplasm worldwide [1]. With more than 700,000 death in year 2018, HCC is considered the third leading cause of cancer-related death [2]. The current treatment of HCC involves some complex decision-making processes clinically such as resection, ablation and etc [3]. Hence, optimized methods of diagnosis and prognosis prediction for the diseases became essential for obtaining a better outcome. Alpha-fetoprotein (AFP) and protein induced by vitamin K antagonist-II (PIVKA-II) are widely appreciated as diagnostic biomarkers; however, a highly effective, universal gene panel for HCC prognosis prediction is yet to be widely adopted [4, 5].

In current clinical practice, the outcome of HCC patients was mainly assessed by models based on tumor pathological characters. The Barcelona Clinic Liver Cancer (BCLC) classification is the most used and verified system for HCC with estimated median survival periods at each tumor stage [6]. Other staging systems proposed by Italian, Japanese and Hong Kong scholars provide alternatives with comparable or enhanced accuracy as BCLC, but further prospective study for validation is needed [7–9].

Nevertheless, all these fail to incorporate molecular markers as prognostic predictive factors. Generally, biological markers are viewed as a pivotal indicator for tumor diagnosis, therapeutic effectiveness, and public tumor surveillance. Not surprisingly, a considerable amount of effort has also been made in developing novel biomarkers or signatures for HCC prognosis over the last decade. For instance, Long et al has developed a four-gene (CENPA, SPP1, MAGEB6, HOXD9) based model to predict the overall survival of HCC patients [10]. Liu et al has also established a four-gene (ACAT1, GOT2, PTDSS2, UCK2) based signature but with genes only in metabolic activity [11]. Besides gene expression, Yang et al has identified the TP53 mutation status also serves as a prognosis indicator for HCC [12].

Despite a great number of researches have done on such genomic indexes, most of them focus on protein coding region and their effect on the patients / disease. To date, the value of non-coding RNAs in HCC prognostic assessment has not been thoroughly explored. In recent years, growing evidence has indicated the crucial role of lncRNA in multiple stages of HCC development including genesis, progression, and recurrence [13–15]. These findings have strongly implied the remarkable potential of lncRNA being the next prognostic indicator for better staging and monitoring of HCC.

**Figure 1:**
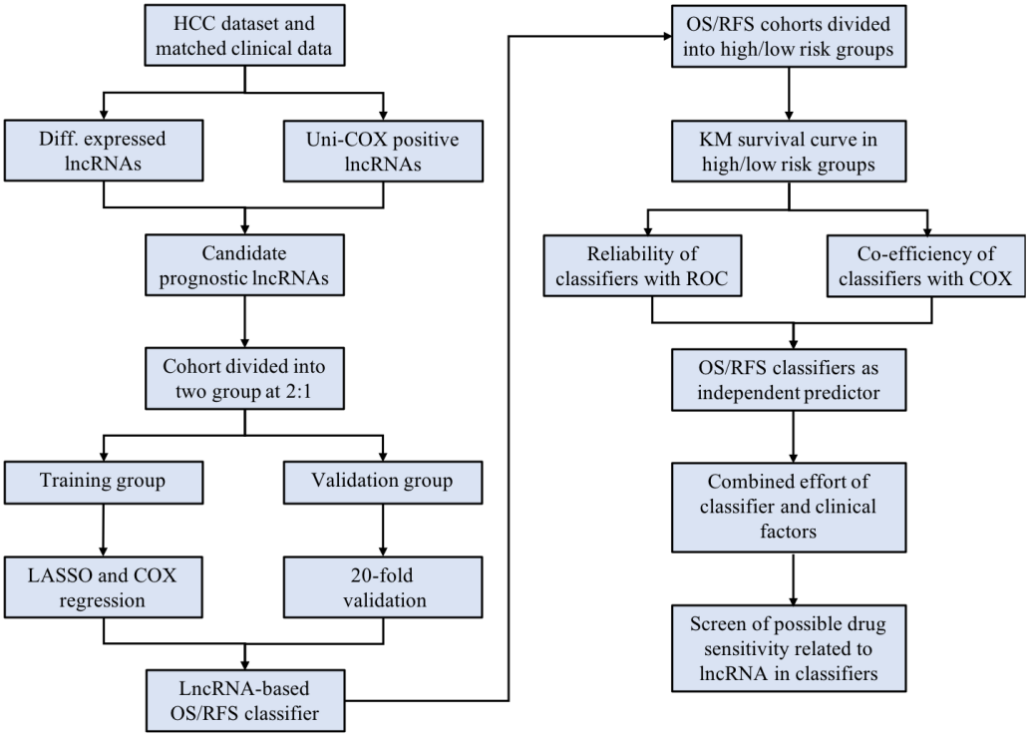
The scheme of the study indicates the major steps of building the lncRNA-based classifiers and following evaluation.

Therefore, this study aims to develop a lncRNA-based tool to monitor and predict the outcomes of HCC. In detail, the HCC cohort containing lncRNA expression and clinical data was acquired from public databases. Two lncRNA-based signatures: an 8-lncRNA contained classifier for overall survival (OS) prediction and a 6-lncRNA contained classifier for relapse-free survival (RFS) prediction, were constructed by applying COX and LASSO regression with differentially expressed lncRNAs (DElncRNAs). Subsequently, the ability as prognostic predictors of both classifiers was evaluated and compared with traditional staging systems. Last but not least, as the cohort was divided into high- and low-risk groups according to the risk score determined by the signature classifiers, potential therapeutic targets and small molecules for high-risk, poor prognosis-associated patients were explored with methods described below (Figure 1).

## RESULTS

### Identification of candidate prognostic lncRNAs

**Figure 2:**
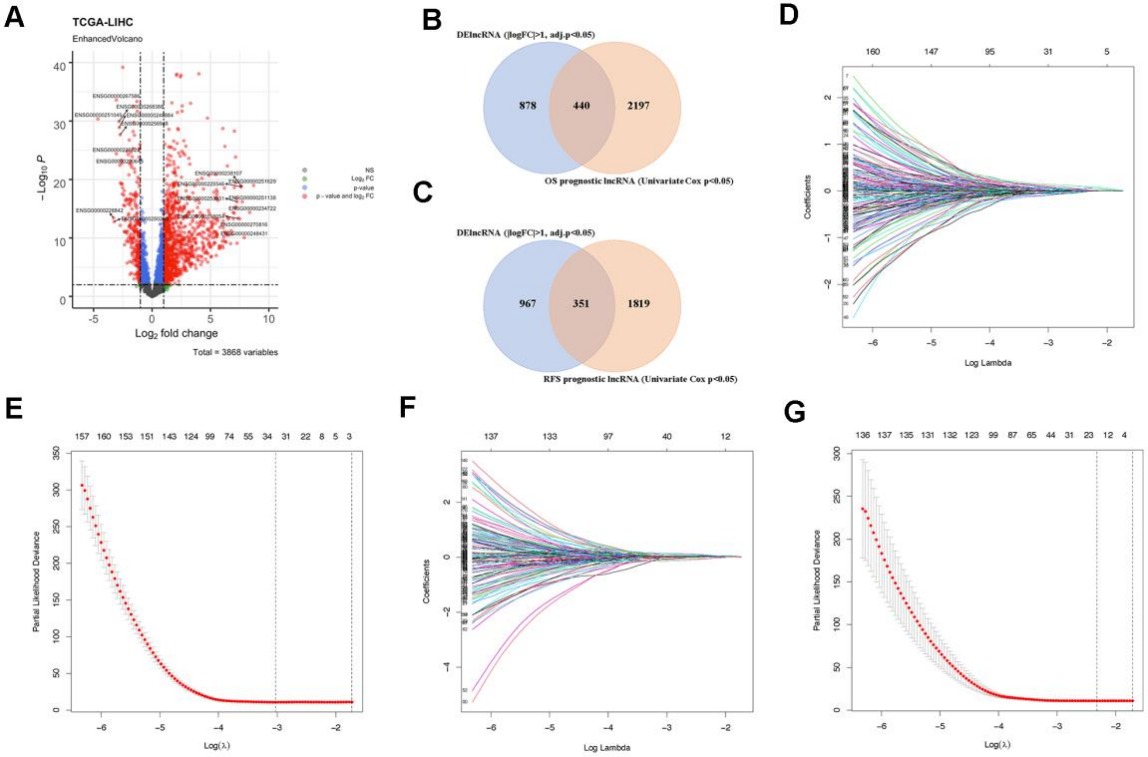
**Identification of prognostic lncRNAs.** (**A**) Volcano plot showing DElncRNAs identified from the TCGA-LIHC dataset. (**B**) Venn diagram of prognostic DElncRNAs obtained from crossing DElncRNAs and COX positive lncRNAs in the OS cohort. (**C**) Venn diagram of prognostic DElncRNAs obtained from crossing DElncRNAs and COX positive lncRNAs in the RFS cohort. (**D**) LASSO regression in the OS cohort according to Lambda value. (**E**) The coefficient profiles of prognostic DElncRNAs in the OS cohort. (**F**) LASSO regression in the RFS cohort according to Lambda value. (**G**) The coefficient profiles of prognostic DElncRNAs in the RFS cohort.

The comprehensive RNA expression profile containing tumor tissue (n = 369) and adjacent control (n = 50) was accessed from the TCGA database as previously described. Of the 14089 lncRNAs extracted from the RNA-seq data, 1318 lncRNAs were identified as DElncRNA under the condition of |logFC| > 1 and adj.p < 0.05 (Figure 2A and Supplementary Figure 1). Besides, 2637 and 2170 lncRNAs related to OS and RFS duration time were screened out by univariate COX regression analysis (p < 0.05). Subsequently, prognostic gene candidates for OS (n = 440) and RFS (n = 351) were determined by overlapping DElncRNAs and univariate COX positive lncRNAs (Figure 2B, 2C). The training cohort and the validation cohort for both OS and RFS classifiers do not have a significant difference. The LASSO regression and multivariate COX analysis were then performed in OS and RFS training group, respectively, at a 20-fold cross-validation manner to generate the lncRNA-based classifiers for OS (Figure 2D, 2E and Supplementary Figure 2A), and RFS (Figure 2F, 2G and Supplementary Figure 2B) prognostics.

### Construction of OS and RFS prediction classifiers

According to the screening process listed above, an 8-lncRNAs-based classifier for OS prediction and a 6-lncRNAs-based classifier for RFS prediction were constructed. The information of the elemental lncRNAs was listed in detail (Table 1).

**Table 1:** The detailed information of lncRNAs in OS- and RFS- classifiers.

**8 lncRNA-based classifier for OS**				
**Gene ID**	**Gene name**	**Chromosome**	**Start point**	**End point**
ENSG00000230587	LINC02580	2p21	43070403	43143114
ENSG00000234899	SOX9-AS1	17q24.3	72040713	72237203
ENSG00000245248	USP2-AS1	11q23.3	119364359	119527977
ENSG00000246985	SOCS2-AS1	12q22	93503696	93571768
ENSG00000254340	AC022784.5	8p23.1	9137584	9145503
ENSG00000261012	AC115619.1	2p24.1	20999312	21000917
ENSG00000262136	AC092115.3	16q22.1	69726533	69742563
ENSG00000267583	AC007998.3	18q12.2	35435198	35467165
**6 lncRNA-based classifier for RFS**				
**Gene ID**	**Gene name**	**Chromosome**	**Start point**	**End point**
ENSG00000223393	AL118511.1	1q42.2	230868259	230879141
ENSG00000254333	NDST1-AS1	5q33.1	150474817	150486291
ENSG00000255571	LINC00925	15q26.1	89361578	89398605
ENSG00000262823	AC127521.1	17p13.2	4480379	4486452
ENSG00000267905	AC008750.2	19q13.41	51340020	51345050
ENSG00000270547	LINC01235	9p23	13404750	13488226

**Figure 3:**
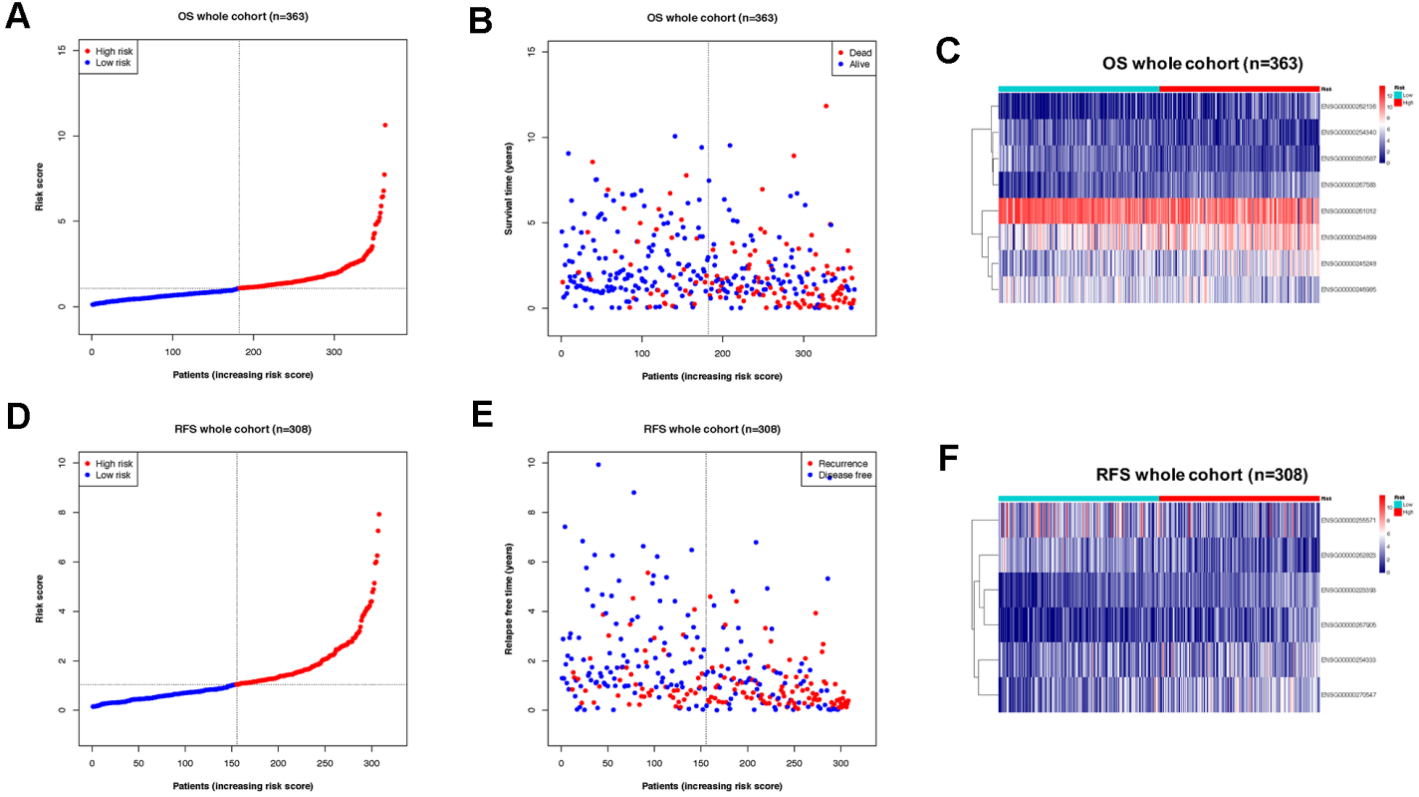
**Division of OS/RFS cohorts into sub-groups by risk score of lncRNA-based classifiers.** (**A**) Distribution of patients the OS whole cohort according to risk score by the classifier. (**B**) Sub-groups in OS cohorts with different vital status. (**C**) Expression of lncRNAs from the OS classifier in high- and low-risk groups of the OS cohort. (**D**) Distribution of patients the RFS whole cohort according to risk score by the classifier. (**E**) Sub-groups in RFS cohorts with different recurrence status. (**F**) Expression of lncRNAs from the RFS classifier in high- and low-risk groups of the RFS cohort.

Subsequently, the OS cohort was further divided into two sub-groups (high-risk and low-risk) according to the median value of the risk score calculated by the OS classifier. The distribution of risk scores, the vital status of patients, and expression of element lncRNAs were compared between high-risk and low-risk subgroups of the OS cohort (Figure 3A–3C). The features of the risk score determined by the RFS classifier in the RFS cohort were also shown in a similar manner (Figure 3D–3F).

**Figure 4:**
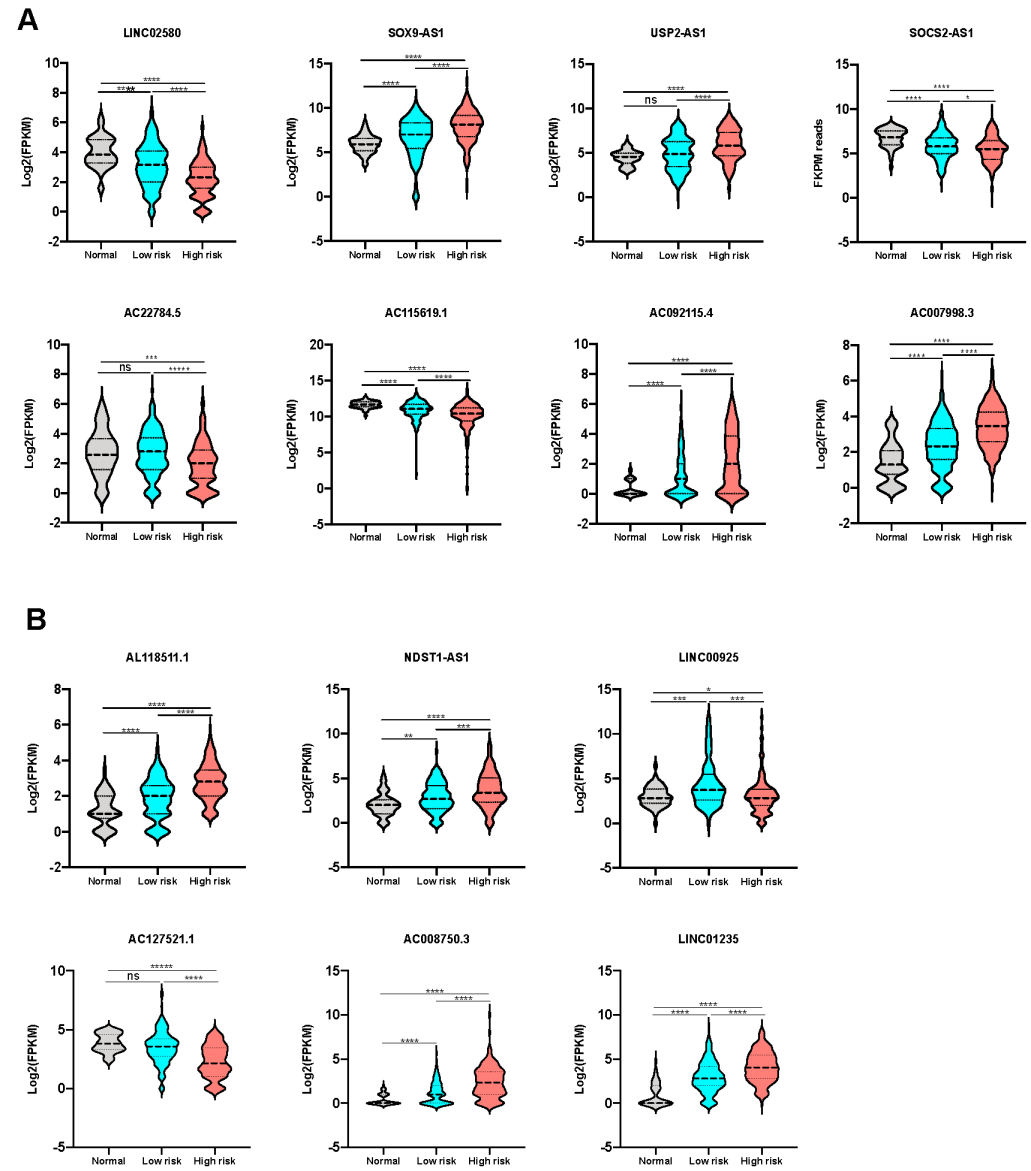
**Expression level of lncRNAs from the signatures in different sub-groups.** (**A**) The expression level of lncRNAs consisting OS classifier in control, low-risk and high-risk groups. (**B**) The expression level of lncRNAs consisting RFS classifier in control, low-risk and high-risk groups.

The expression of the lncRNAs from both prognostic classifiers was then compared in the high-risk group, low-risk group, and non-tumor control group, to confirm the differential expression level between the high risk and low risk group. As expected, all lncRNAs for OS prediction (Figure 4A) and RFS prediction (Figure 4B) showed a significant differential expression between the high-risk group and low-risk group, further validating the hypothesis that the expression of these prognostic lncRNAs could be correlated to tumor progression in HCC.

### Assessment of the lncRNA signatures for HCC prognosis prediction

**Figure 5:**
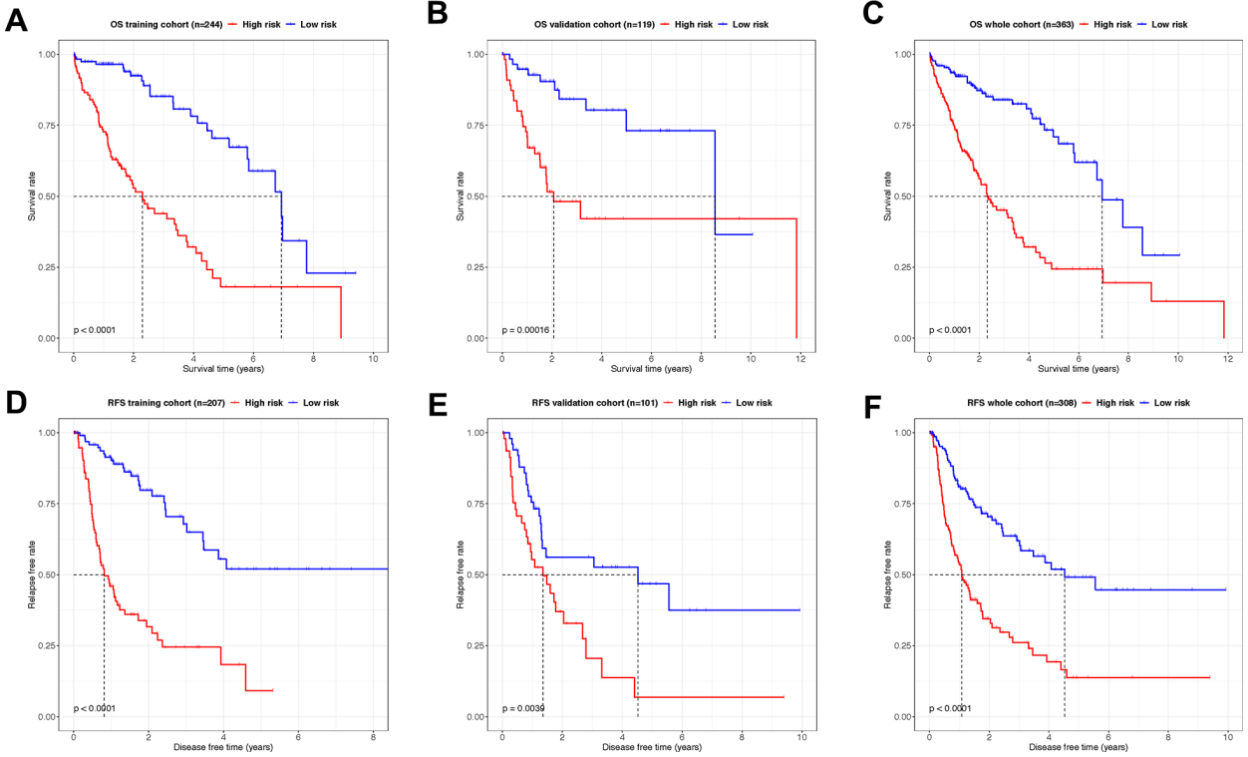
**Kaplan–Meier analysis showing the OS- and RFS-time expectancy.** (**A**–**C**) The overall survival curves of HCC patients in training, validation and whole cohorts grouped by the risk level. (**D**–**F**) The relapse-free survival curves of HCC patients in training, validation and whole cohorts grouped by the risk level.

The predictive capacity of both OS- and RFS signatures were evaluated in all the training, validation, and whole cohorts, respectively. Kaplan-Meier log-rank tests were conducted in all 6 groups to confirm the effectiveness and consistency of the model for both OS (Figure 5A–5C) and RFS (Figure 5D–5F) prediction. Unanimously, in all the cohorts with OS and RFS prognostic panels, patients in high-risk groups showed significantly poorer outcomes of either demise or tumor relapse (P < 0.01). These results indicated that the OS- and RFS-classifiers significantly linked with the prognosis of HCC, thus hold the potential as an effective prediction model.

**Figure 6:**
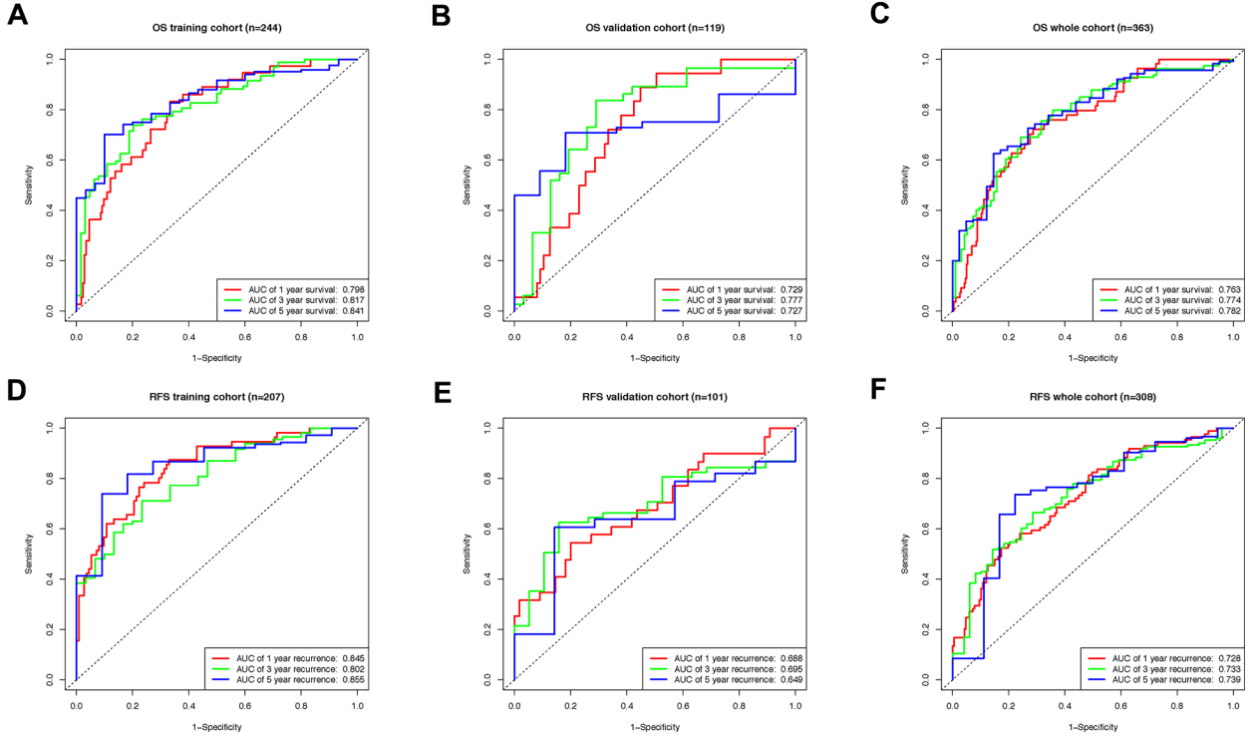
**The time-dependent ROC curve evaluating the efficiency of lncRNA based classifiers.** (**A**) The ROC curve indicating the efficiency of lncRNA-based classifier as OS prognosis indicator in training, (**B**) validation, (**C**) and whole groups. (**D**) The ROC curve indicating the efficiency of lncRNA-based classifier as RFS prognosis indicator in training, (**E**) validation, (**F**) and whole groups.

Afterward, the efficiency of both classifiers was checked by the time-dependent receiver operating characteristic (ROC) curve. In the OS cohort, areas under ROC curve (AUCs) of the 8-lncRNA-based classifier reached 0.798, 0.817 and 0.841 for 1, 3, and 5 years in the training group (Figure 6A), 0.729, 0.777 and 0.727 in the validation group (Figure 6B), 0.763, 0.774 and 0.782 for 1, 3, and 5 years in the whole group (Figure 6C). Meanwhile, the AUCs of the 6-lncRNA-based classifier for RFS prediction were 0.845, 0.802, 0.855. for 1, 3, and 5 years in the training group (Figure 6D), 0.688, 0.695, 0.649 in the validation group (Figure 6E), 0.728, 0.733, 0.739 in the whole RFS group (Figure 6F).

### Comprehensive prognostic analysis of lncRNA signatures and clinical pathological characteristics

As described above, the lncRNA-based signatures were proved to be a prognosis indicator with high accuracy in predicting the outcomes of both OS and RFS time for HCC patients. However, whether the risk score of the novel lncRNA-based classifiers is correlated with other clinicopathologic characteristics requires further exploration.

Hence, some major clinical factors were listed and compared with the risk score with Pearson chi-square test in the OS- and RFS-cohorts, separately (Table 2). The analysis indicated that in the OS cohort, pT and tumor stage were significantly associated with risk score levels. In the RFS cohort, pT and tumor stage also showed relevance with the risk score levels despite less significance. In all, a high-risk score level often implies late pT, higher tumor stage, and after all, short overall survival and relapse-free survival time.

**Table 2:** Correlations between risk score of the OS- and RFS-classifiers and clinicopathological characteristics.

**Clinicopathologic features in the OS cohorts**				
**Variable**	**High risk**	**Low risk**	**Pearson x2**	**P-value**
**Age**				
**>60**	97	94	0.9914	0.7529
**=<60**	85	88		
**Gender**				
**male**	122	123	0.01248	0.9110
**female**	60	59		
**pT**				
**T3-T4**	75	37	18.62	**0.0001**
**T0-T2**	107	145		
**pN**				
**N1-N3**	61	56	0.3149	0.5747
**N0**	121	126		
**pM**				
**M1**	49	53	0.2179	0.6406
**M0**	133	129		
**Tumor stage**				
**Stage III-IV**	79	36	23.5	**0.0001**
**Stage I-II**	103	146		
**Clinicopathologic features in the RFS cohorts**				
**Variable**	**High risk**	**Low risk**	**Pearson x2**	**P value**
**Age**				
**>60**	82	78	0.2067	0.6494
**=<60**	73	77		
**Gender**				
**male**	101	105	0.2315	0.6304
**female**	54	50		
**pT**				
**T3-T4**	44	28	4.631	**0.0314**
**T0-T2**	111	127		
**pN**				
**N1-N3**	44	42	0.06437	0.7997
**N0**	111	113		
**pM**				
**M1**	32	39	0.8952	0.3441
**M0**	123	116		
**Tumor stage**				
**Stage III-IV**	50	29	7.491	**0.0062**
**Stage I-II**	105	126		

To compare the efficiency of lncRNA classifiers and other prognostic factors, age, gender, pT, pN, pM, tumor stage and lncRNA-based classifiers were assessed by a two-step COX regression analysis (Table 3). In OS cohorts, pT, pM, tumor stage and risk score defined by the 8-lncRNA-based classifier were found significantly associated with OS-time in the univariate COX test. Interestingly, only the risk score and pM remained positive as the independent predictor in all the OS groups after multivariate COX analysis, while the risk score revealed a dramatically higher efficiency than pM. Similar in RFS groups, pT, tumor stage, and the 6-lncRNA-based classifier were positively related to RFS-time in univariate COX analysis, yet only the risk score of the RFS lncRNA classifier remained significant in the following multivariate COX with high efficiency.

**Table 3:** Uni-and multivariate COX regression of the prognostic factors for OS and RFS prediction.

**8-lncRNA-based OS classifier**				
**Parameter**	**Univariate COX**	**P value**	**Multivariate COX**	**P value**
	**HR (95% CI)**		**HR (95% CI)**	
**Age (> 60 vs ≤ 60)**	1.01(1.00-1.02)	0.175520599		
**Gender (male vs female)**	1.22(0.85-1.75)	0.2775024378		
**pT (3-4 vs 0-2)**	1.65(1.37-2.00)	**0.0000001391**	1.77(0.95-3.28)	0.070485113
**pN (1-3 vs 0)**	1.42(0.98-2.05)	0.0644260708		
**pM (1 vs 0)**	1.73(1.19-2.51)	**0.0037449858**	1.78(1.22-2.58)	**0.002747801**
**Stage (III-IV vs I-II)**	1.66(1.36-2.02)	**0.0000005323**	0.75(0.39-1.43)	0.376683648
**Risk score (high vs low)**	1.40(1.29-1.53)	**0.0000000001**	4.01(2.66-6.05)	**0.0000000001**
**6-lncRNA-based RFS classifier**				
**Parameter**	**Univariate COX**	**P value**	**Multivariate COX**	**P value**
	**HR (95% CI)**		**HR (95% CI)**	
**Age (> 60 vs ≤ 60)**	1.00(0.98-1.01)	0.6332914949		
**Gender (male vs female)**	0.89(0.62-1.27)	0.5226851602		
**pT (3-4 vs 0-2)**	1.64(1.36-1.97)	**0.0000001770**	1.77(0.63-4.98)	0.2754281773
**pN (1-3 vs 0)** **pM (1 vs 0)**	1.17(0.80-1.69) 1.20(0.82-1.76	0.4162533310 0.3502956657		
**Stage (III-IV vs I-II)**	1.64(1.36-1.99)	**0.0000004245**	0.80(0.27-2.33)	0.6824451077
**Risk score (high vs low)**	1.73(1.55-1.93)	**0.0000000001**	1.45(1.27-1.66)	**0.0000000537**

**Figure 7:**
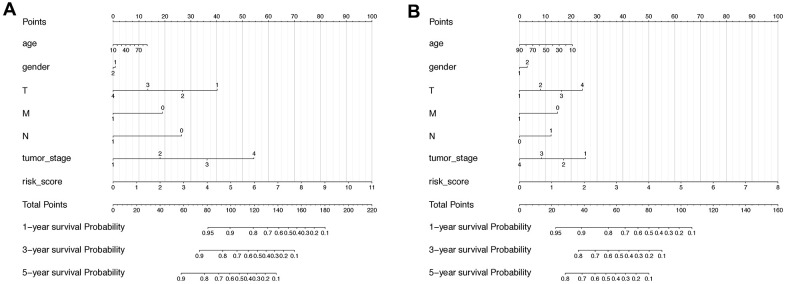
**Nomogram including lncRNA-based signature and other pathoclinical factors for both OS and RFS prognosis prediction.** (**A**) Nomogram including risk score determined by the lncRNA-based signature and other pathoclinical factors for OS prognostic assessment of HCC. (**B**) Nomogram including risk score determined by the lncRNA-based signature and other pathoclinical factors for RFS prognostic assessment of HCC.

As the lncRNA signatures and several pathological features concordantly showed significant correlation with HCC progression, their combined efforts in predicting HCC prognosis were further check with the Normogram analysis. In specific, the risk score is the most relevant indicator in the diagram with the total points reflecting the final prognostic probability in OS of HCC, while the T status also plays a critical role (Figure 7A). In RFS prognosis, despite the risk score of the lncRNA classifier and the T status remained the major anchor for prognosis, factors such as Age and Tumor stage were interestingly gained more weight on deciding total probability points compared with OS prognosis (Figure 7B).

Taking together, the lncRNA-based classifiers could be considered as an independent indicator for both OS and RFS prediction of HCC with high efficiency.

### Identification of potential small molecules for high-risk score patients

**Figure 8:**
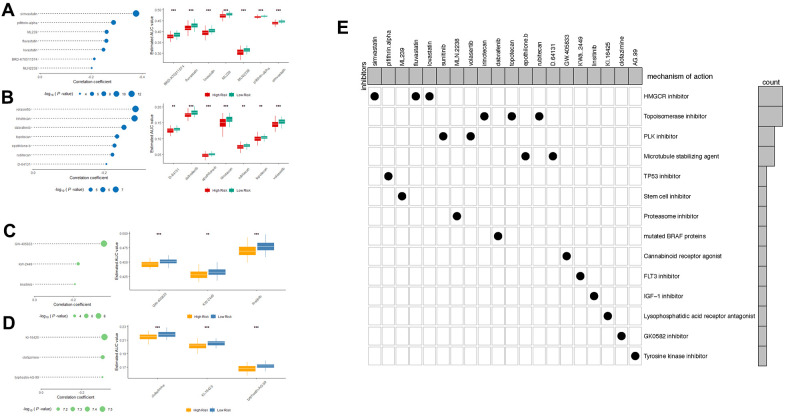
Spearman’s correlation analysis and differential drug response analysis of 7 CTRP-derived compounds; (**A**) and 7 PRISM-derived compounds (**B**) with OS-classifier; (**C**) and 3 PRISM-derived compounds; (**D**) with OS-classifier. Note that lower values on the y-axis of boxplots imply greater drug sensitivity. (**E**) Heatmap showing each compound (perturbagen) from the CMap dataset that shares mechanisms of action (rows) and sorted by descending number of compound with shared mechanisms of action.

To identify drug candidates for high-risk patients with our LncRNA signature, two different approaches were applied using CTRP and PRISM drug response database separately. The differential drug sensitivity was identified between high- (top 20%) and low-risk (bottom 20%) patients with lower estimated AUC values in high-risk patients (Log2FC >0.05). Following this, the spearman correlation was measured to select the candidates with a negative correlation coefficient (r < -0.2) between risk scores and AUC values with both OS and RFS signatures. With the OS- signature, seven candidates from CTRP and seven candidates from the PRISM dataset were identified (Figure 8A, 8B). And applying the RFS signature, a total of six compounds were screened out from both datasets using the same criteria (Figure 8C, 8D). All candidates were having significantly lower estimated AUC values in high-risk patients. To further investigate the mechanism of these drug candidates, the Cmap mode-of-action (MoA) database including nearly 3000 small-molecule compounds was applied (Figure 8E). The target analysis revealed 14 distinct drug targets in those candidates, and the top enriched targets are HMGCR inhibitors and topoisomerase inhibitors.

## DISCUSSION

As HCC, the primary malignancy in the liver, remains high frequency of recurrence and mortality despite a comprehensive treatment pool and protocol including radical resection, ablation, and recently arisen targeted- or immunotherapy. For many years, physicians and scientists solely relied on models limited in pathological classification such as TNM phase and tumor stages to predict the outcomes of HCC and making clinical decisions. However, the diverse background disease and heterogenetic nature make the accurate prediction of HCC prognosis exceedingly challenging. Under the circumstance, a novel biomarker panel could be an alternative concept and beneficial for clinical surveillance and management of HCC.

In recent years, numerous pieces of evidence have shown the critical role of non-coding RNAs including miRNA, circRNA and especially lncRNA in extensive biological processes. Studies have reported that lncRNAs function as master regulators of gene transcription, mRNA processing, and nucleus modification [16]. Dysregulated ncRNAs also contribute to pathological processes such as carcinogenesis and metastasis of different malignant diseases, HCC included. Nevertheless, only a limited number of lncRNAs have been extensively studied in HCC while the role of the rest majority remains largely obscure. For instance, the lncRNA XIST manipulating X chromosome inactivation was among one of the earliest investigated lncRNAs [17]. According to a series of recent reports, XIST is down-regulated in HCC thus serves as a tumor suppressor via inhibiting oncogenic miR-497 via the competing endogenous RNA mechanism [18]. In contrast, lncRNA MALAT-1 and HULC were found up-regulated in HCC and promote tumor growth, metastasis and drug resistance by interacting with several pathways closely relevant in HCC progression [19]. To note, the diagnostic and therapeutic potential of lncRNA in HCC has being aware during the past decade [20, 21].

In this study, one large HCC cohort (TCGA-LIHC) was split into training and validation sub-groups with the cross-validation strategy to ensure the stability of the predictive ability. Moreover, the LASSO and COX regression analyses were applied to optimize the selection of candidate lncRNAs with both high expression variances and prognostic values. Last but not least, tumor recurrence is found in more than 60% of HCC patients within 5 years, which reflects the poor prognosis with the progression of the disease [22]. Therefore, the lncRNA-based signature indicating RFS time is of great significance and provides an adequate complement for all-round prediction of HCC prognosis.

After the establishment of signature, individual samples in overlapped OS and RFS cohorts were automatically endowed with a risk score with both OS and RFS classifier. Patients with high and low-risk scores revealed a significant difference in overall and relapse-free life expectancy according to Kaplan-Meier curves. In addition, pT, pM, tumor stage and lncRNA-based classifier are all correlated with OS in univariate COX analysis. However, the 8-lncRNA-based classifier remains relevant with remarkably high efficiency in the following multivariate COX regression analysis compared to other models. Similarly, the pT, tumor stage and 6-lncRNA-based classifier are related to RFS in univariate COX but only the lncRNA classifier remains the sole significant indicator in the multivariate COX regression model. Additionally, the classifiers exhibit superior accuracy in prognostic prediction, with AUCs exceeding 0.75 in all 1, 3, and 5 years timepoint for OS prediction, and also reaching over 0.7 for RFS prediction. In comparison, the AUCs of tumor stage as a predictor are only approximate 0.6, apparently inferior to lncRNA-based classifier in both cases (Supplementary Figure 3).

Interestingly, although the two lncRNA-based classifiers are proven to be promising predictors for HCC prognosis, lncRNAs forming the signature remain largely unstudied in tumor biology. To note, the finding that elemental lncRNAs had little mutual correlation in expression suggests they might have different or irrelevant mechanisms (Supplementary Figure 4).

Among all the lncRNAs consisting the OS classifier, LINC02580 was reported strongly down-regulated in HCC compared with normal liver—consist with our study—and low expression of LINC02580 linked with poor prognosis. Gene SRSF1 mediating genetic alternative splicing was likely the target of LNC02580 but the detailed mechanism remained unexplored [23]. Moreover, SOX9-AS1 was shown to form a positive feedback loop with its relative gene SOX9, an oncogenic transcriptional factor, via acting as a sponge for microRNA-5590 [24]. Besides, SOCS2-AS1 was found related (often negatively) to the progression of several cancer types including endometrial cancer, colorectal cancer, prostate cancer, while few reports were seen relating to rest members of the 8-lncRNA-based signature [25–27]. On the other hand, lncRNAs from the RFS classifier received even much less attention compared to their counterparts in the OS classifier. LINC01235 was the sole gene studied in previous literature. Papers have indicated the function of LINC01235 to be a prognostic marker, as well as to promote tumor progression via facilitating epithelial-mesenchymal transition in gastric cancer [28–30]. Therefore, the next step would probably be conducting functional studies to gain deeper understandings of these lncRNAs and to particularly reveal novel mechanisms in HCC development.

To further identify the potential drug targets and candidate small molecules for the high-risk patients, two drug response datasets (CTRP and PRISM) were applied for small molecules screening and the CMap database was supplied with MoA information. The top enriched drug targets are HMGCR inhibitors and topoisomerase inhibitors.

Statins were widely used in patients to lower cholesterol to reduce the risk of a heart attack or stroke. As HMGCR inhibitors, statins were reported associated with reduced risk of HCC development in chronic HBV-infected patients, HCV-infected patients, and diabetes patients [31, 32]. More importantly, patients diagnosed with HCC showed significantly decreased mortality with the treatment of statins. In molecular level studies, HMGCR inhibitors reduced the FoxM1 transcription factor through the mevalonate pathway [33]. Topoisomerase plays important role in cellular proliferation and DNA structure. Topoisomerase inhibitors were often used as cytotoxic chemotherapy drugs in multiple malignancies in the clinic. Previous studies revealed that Irinotecan activates p53 signaling to induce HCC apoptosis [34]. Numbers of studies also focus on the combination therapy strategy with topoisomerase inhibitors. Dasatinib (tyrosine kinase inhibitor) and gefitinib (EGFR inhibitor) showed synergistic effect with irinotecan in HCC models, which implies potential clinical benefit for high-risk patients [35, 36].

In conclusion, this study generated paired novel lncRNA-based signatures to predict both the overall survival and recurrence of HCC. The superior effectiveness and efficiency of the model as independent prognosis indicator have been demonstrated in different manners. The application of the lncRNA-based signature, either alone or in combined efforts with other clinical factors, tend to provide novel solution for improved prognosis anticipation and clinical management of HCC, and eventually benefit both patients and doctors. But before achieving this, more efforts on validation and mechanistic exploration on these genes are still in significant need.

## MATERIALS AND METHODS

### HCC dataset acquisition

An RNA-seq dataset of 371 HCC patients involving RNA sequencing and matched clinical characteristics were obtained from the TCGA data portal (accessed on September 7, 2020). The cohort contains 374 HCC tumor tissues and 50 adjacent liver tissue as control, and the matched clinical information was acquired from Cbioportal (accessed at September 8, 2020) [37]. All data acquisition processes fully complied with TCGA publication policies [38].

### Data processing

Genome-wide all RNA expression was acquired from the TCGA dataset as described above. The data were annotated by the Gencode GTF file (Gencode v35, acquired at http://gencodegenes.org). Then the lncRNA was separated from gene-coding RNA and other non-coding RNAs. lncRNAs with zero counts were excluded. Differentially expressed lncRNAs (DElncRNAs) were identified by the R programming edgeR package with the criteria of |logFC| > 1 and adj.p < 0.05 between tumor and control tissues.

Afterward, we performed a univariate COX regression to screen out lncRNAs that correlated with the clinic OS and RFS time of the patients (p < 0.05). Eventually, the candidate prognostic lncRNAs were determined by overlapping the DElncRNAs and univariate COX positive lncRNAs for further analysis.

### Construction of lncRNA-based prognostic signature

Next, both the OS and RFS cohorts were randomly split into training and validation groups at a 2:1 ratio. The LASSO regression was performed at 20-fold cross-validation in the two training groups to generate an 8-lncRNA-based OS classifier and a 6-lncRNA-based RFS classifier. According to the cut-off median value of the risk score, the OS and RFS cohorts were divided into high- and low-risk groups, then the expression of element lncRNAs within the classifiers in control, low-risk, and high-risk groups were compared.

The predicting capability of the lncRNA classifiers in both training, validation, and whole cohort were subsequently confirmed by the Kaplan-Meier long-rank test, Time-dependent ROC curve analysis, and multivariate COX regression. All the analyses were conducted using GraphPad Prism 7 and R platform version 4.0.2 with packages ‘edgeR’, ‘carnet’, ‘survmine’, ‘glmnet’, and ‘ROCR’.

### Drug sensitivity screening and mechanism of actions analysis

Drug sensitivity data of human cancer cell lines were achieved from the Cancer Therapeutics Response Portal (CTRP v2, Board institute) and PRISM repurposing dataset (https://depmap.org/repurposing/). The algorism of drug sensitivity was described in previous studies [12]. Briefly, the two databases provided AUC (area under the curve) as the readout of drug sensitivity. The lower AUC values indicate higher drug sensitivity. Compounds with more than 20% missing data were excluded from the dataset, and the K-nearest neighbor algorithm (k-NN) was applied to estimate the AUC values. To further investigate the mechanism of actions (MoA) of the drugs screened out, the Connectivity Map tools database (https://clue.io/) with 2429 small molecules perturbagen types was applied for specific analysis [39].

## Supplementary Material

Supplementary Figures
