# Supplementary material for: Identification of prognostic long non-coding RNA signature with potential drugs in hepatocellular carcinoma
Source: Aging (Albany NY). 2021 Jul 20;13(14):18789–805. doi: 10.18632/aging.203322 (PMC8351707; doi:10.18632/aging.203322)
Supplement: Supplementary Figures [file aging-13-203322-s001.pdf]

SUPPLEMENTARY FIGURES

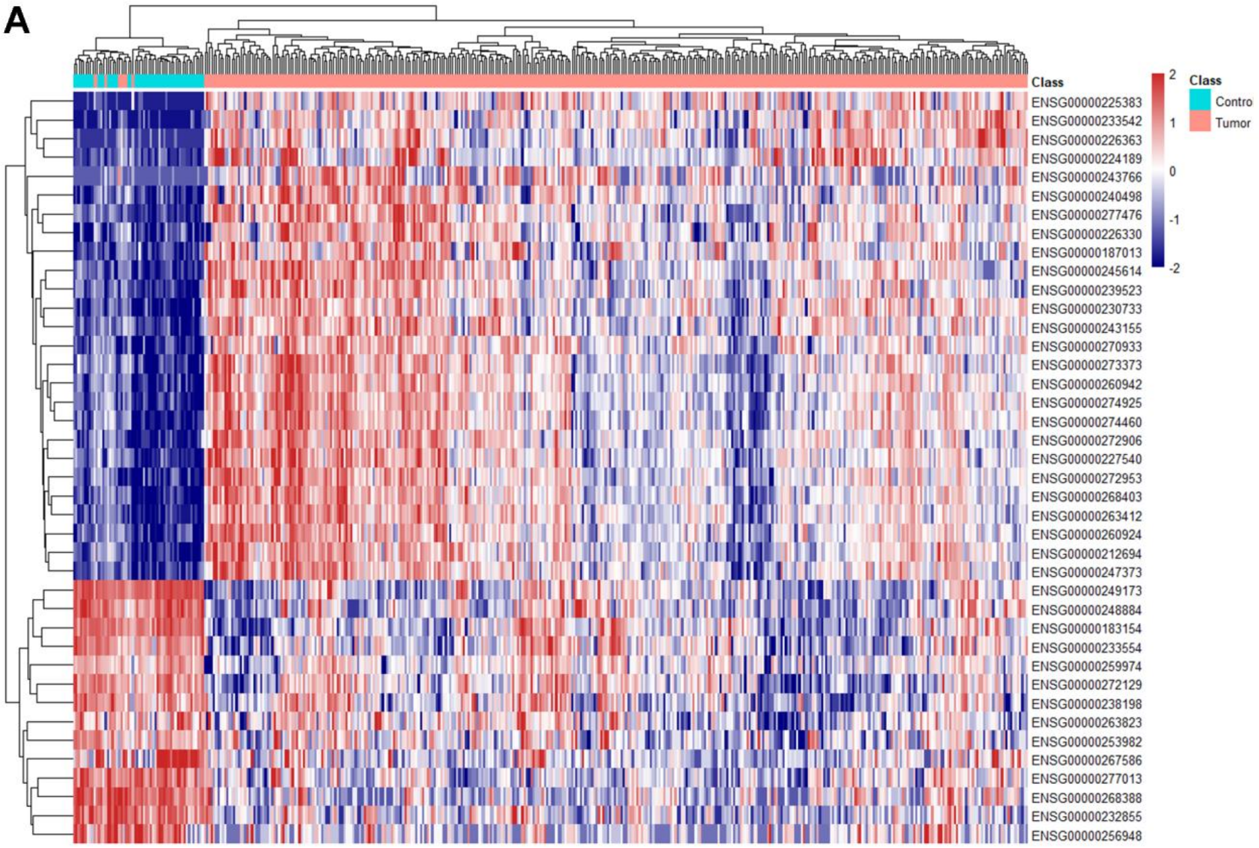

Supplementary Figure 1. Heatmap of differentially expressed lncRNAs in TCGA-LIHC cohort. (**A**) Heatmap showing 20 most up-regulate lncRNAs and 20 most down-regulated lncRNAs in TCGA-LIHC cohort (control tissue vs. tumor) by adjust p value.

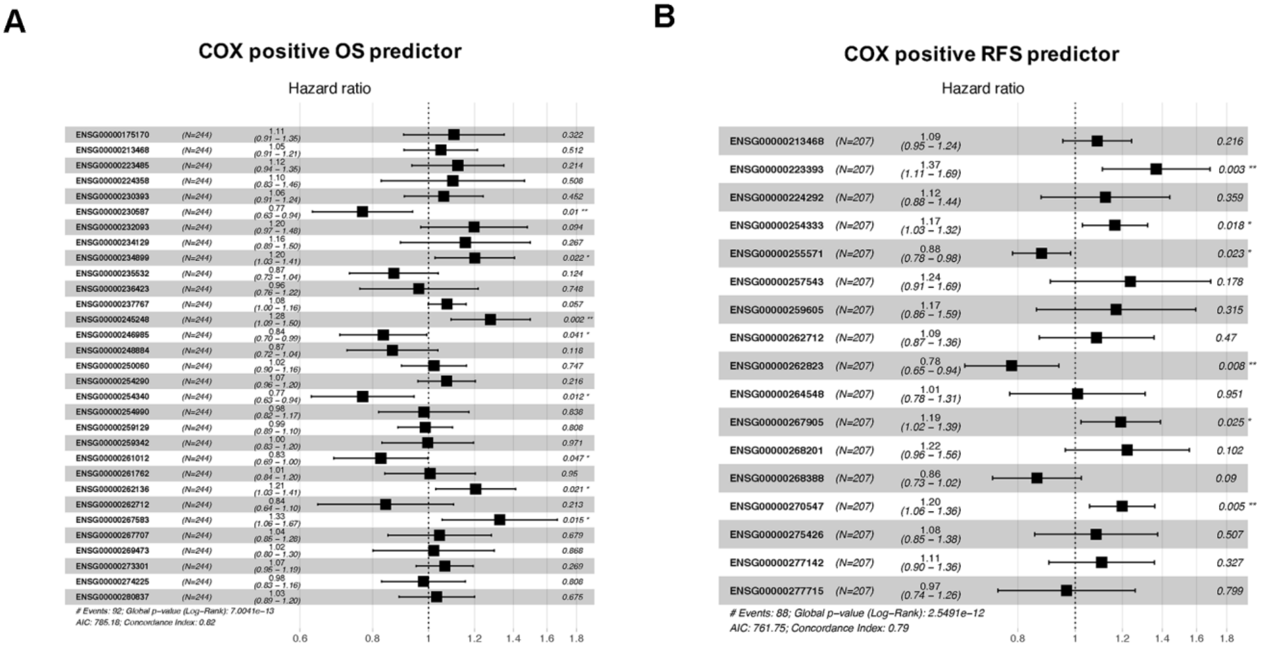

Supplementary Figure 2. COX regression analysis indicating candidate lncRNAs for prognostic signature. (**A**) COX regression analysis selecting prognostic candidate from LASSO-positive lncRNAs in OS cohort. (**B**) COX regression analysis selecting prognostic candidate from LASSO-positive lncRNAs in RFS cohort.

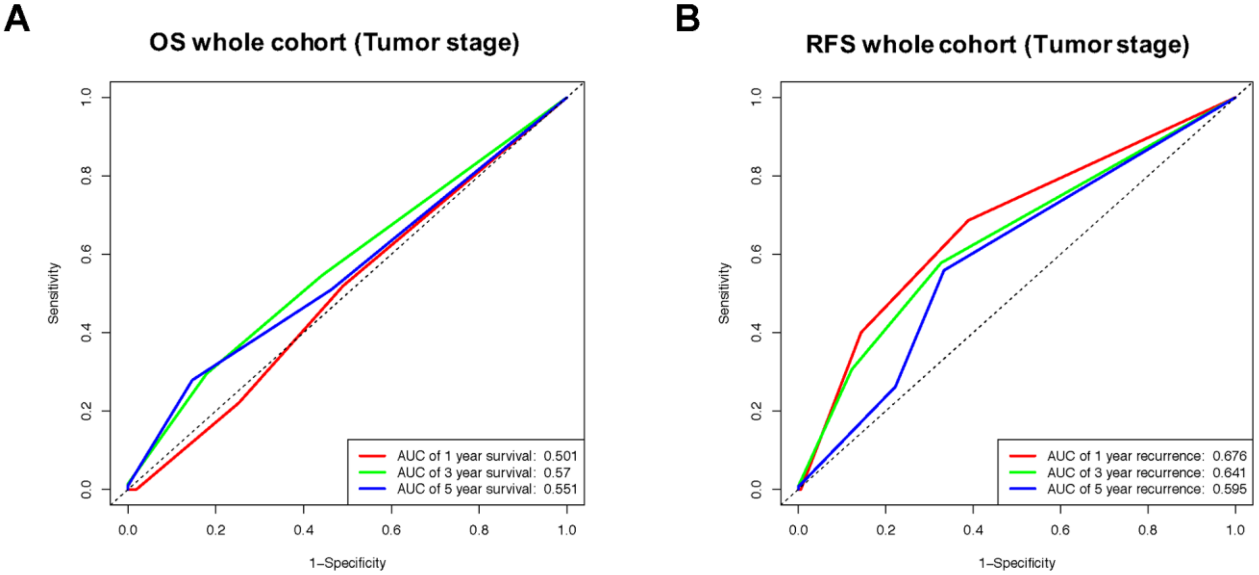

Supplementary Figure 3. The time-dependent ROC curve evaluating the efficiency of tumor stage as prognostic indicator. (**A**) The ROC curves showing tumor stage for OS prognosis assessment. (**B**) The ROC curves showing tumor stage for RFS prognosis assessment.

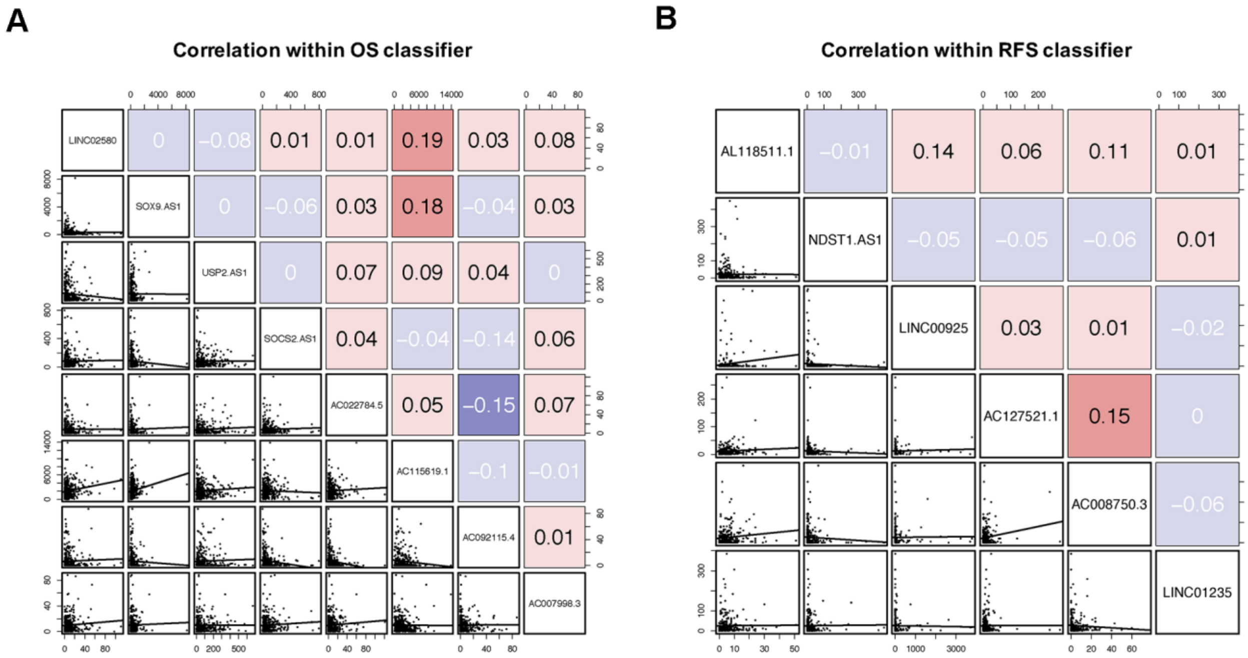

Supplementary Figure 4. Expressional correlation of member lncRNAs in lncRNA-based classifiers. (**A**) Correlation of 8 lncRNAs consisting OS prognosis signature. (**B**) Correlation of 6 lncRNAs consisting RFS prognosis signature.
